# Substance P-expressing Neurons in the Superficial Dorsal Horn of the Mouse Spinal Cord: Insights into Their Functions and their Roles in Synaptic Circuits

**DOI:** 10.1016/j.neuroscience.2020.06.038

**Published:** 2020-12-01

**Authors:** Erika Polgár, Andrew M. Bell, Maria Gutierrez-Mecinas, Allen C. Dickie, Oğuz Akar, Miruna Costreie, Masahiko Watanabe, Andrew J. Todd

**Affiliations:** a Institute of Neuroscience and Psychology, College of Medical, Veterinary and Life Sciences, University of Glasgow, Glasgow G12 8QQ, UK; b Department of Anatomy, Hokkaido University School of Medicine, Sapporo 060-8638, Japan

**Keywords:** AAV, adeno-associated virus, ALT, anterolateral tract, CCK, cholecystokinin, CTb, cholera toxin B subunit, eGFP, enhanced green fluorescent protein, GRP, gastrin releasing peptide, LPb, lateral parabrachial area, LSN, lateral spinal nucleus, NKB, neurokinin B, NPFF, neuropeptide FF, SDH, superficial dorsal horn, SP, substance P, TeLC, tetanus toxin light chain, TFP, teal fluorescent protein, brainbow, Tac1, homer, gastrin releasing peptide, spinoparabrachial, tetanus toxin light chain

## Abstract

The tachykinin peptide substance P (SP) is expressed by many interneurons and some projection neurons in the superficial dorsal horn of the spinal cord. We have recently shown that SP-expressing excitatory interneurons in lamina II correspond largely to a morphological class known as radial cells. However, little is known about their function, or their synaptic connectivity. Here we use a modification of the Brainbow technique to define the excitatory synaptic input to SP radial cells. We show that around half of their excitatory synapses (identified by expression of Homer) are from boutons with VGLUT2, which are likely to originate mainly from local interneurons. The remaining synapses presumably include primary afferents, which generally have very low levels of VGLUT2. Our results also suggest that the SP cells are preferentially innervated by a population of excitatory interneurons defined by expression of green fluorescent protein under control of the gene for gastrin-releasing peptide, and that they receive sparser input from other types of excitatory interneuron. We show that around 40% of lamina I projection neurons express *Tac1*, the gene encoding substance P. Finally, we show that silencing Tac1-expressing cells in the dorsal horn results in a significant reduction in reflex responses to cold and radiant heat, but does not affect withdrawal to von Frey hairs, or chloroquine-evoked itch.

## Highlights

• Substance P-expressing radial cells in lamina II receive half of their excitatory synaptic input from other interneurons.
• They are preferentially innervated by transient central cells that express eGFP in a GRP-eGFP mouse line.
• Around 40% of projection neurons in lamina I express Tac1, the gene for substance P.
• Silencing Tac1 cells in the dorsal horn reduces reflex responses to cold and radiant heat.

## Introduction

The neuropeptide substance P (SP) was identified in a plexus of axons in the superficial dorsal horn (SDH; laminae I-II) by Hokfelt et al. (1975), who showed that some of these axons originated from a population of SP-expressing primary afferent neurons. A series of studies involving Tom Jessell published over the next few years demonstrated that SP was located in axon terminals in the dorsal horn (Cuello et al., 1977), that it could be released in response to capsaicin (Theriault et al., 1979), and that it was substantially depleted from the dorsal horn by treatment with capsaicin, as well as by peripheral nerve section or dorsal rhizotomy (Jessell et al., 1978, Jessell et al., 1979, Yaksh et al., 1979). However, following rhizotomy (which should destroy central terminals of primary afferents), the SP concentration in the ipsilateral dorsal horn was ∼20% of that seen in unoperated animals (Jessell et al., 1979), suggesting that some of the peptide is derived from non-primary sources. Subsequent *in situ* hybridisation studies (Warden and Young, 1988, Xu et al., 2013) revealed a population of cells in the SDH with mRNA for *Tac1*, the gene that encodes SP, and SP-immunoreactive cells were identified in this region following treatment with colchicine, to block axoplasmic transport (Hokfelt et al., 1977, Hunt et al., 1981, Nahin, 1987, Leah et al., 1988, Senba et al., 1988, Yoshida et al., 1990, Ribeiro-da-Silva et al., 1991, Battaglia and Rustioni, 1992). It has since been shown that most SP-expressing dorsal horn cells are excitatory interneurons (Gutierrez-Mecinas et al., 2018, Dickie et al., 2019), with some being projection neurons that belong to the anterolateral tract (ALT) (Gutierrez-Mecinas et al., 2018, Huang et al., 2019). Recent transcriptomic studies have identified specific populations among the excitatory interneurons that correspond to SP-expressing cells (Häring et al., 2018, Sathyamurthy et al., 2018). In a series of recent studies (Gutierrez-Mecinas et al., 2017, Gutierrez-Mecinas et al., 2018, Dickie et al., 2019), we have shown that SP-positive excitatory interneurons are particularly numerous in lamina II, that many of them correspond to a population that had previously been defined by Grudt and Perl (2002) as radial cells, and that they can give rise to long propriospinal projections that travel in the dorsolateral fasciculus and target the lateral spinal nucleus (LSN). Consistent with the transcriptomic data, we also showed that they are distinct from other neurochemical populations of excitatory interneurons, which can be defined by expression of neurotensin, neurokinin B (NKB), cholecystokinin (CCK) and neuropeptide FF (NPFF) (Gutierrez-Mecinas et al., 2019a, Gutierrez-Mecinas et al., 2019b). In addition, they do not overlap significantly with cells that contain enhanced green fluorescent protein (eGFP) in the GRP::eGFP mouse line, in which eGFP is expressed under control of the promoter for gastrin-releasing peptide (GRP) (Dickie et al., 2019; Bell et al., 2020).

Although Lu and colleagues (Lu and Perl, 2005, Zheng et al., 2010, Lu et al., 2013) have identified certain synaptic circuits involving other types of excitatory interneuron in lamina II, little is apparently known about the synaptic inputs to the SP-expressing radial cells. We have recently examined the excitatory synapses on a different population, the GRP-eGFP cells (which correspond to the transient central class of Grudt and Perl), and found that these are dominated by primary afferents, with excitatory interneurons apparently providing only ∼10% of the input (Bell et al., 2020). The first aim of the present study was to examine excitatory synaptic input to the SP cells and determine whether this differed from that on the GRP-eGFP cells. In particular, we looked for input from 4 different excitatory interneuron populations: those that expressed neurotensin, NKB or SP, and those originating from the GRP-eGFP cells (Gutierrez-Mecinas et al., 2016a, Dickie et al., 2019 Bell et al., 2020). As well as being expressed by interneurons, SP is also present in some ALT cells in lamina I. However, the proportion of lamina I ALT cells that express SP is not known, and our second aim was to determine this. Finally, it has been reported that while ablation of SP-expressing dorsal horn cells reduces behavioural responses to sustained pain, it does not affect acute nocifensive reflexes (Huang et al., 2019). This is a somewhat surprising observation, given that ∼20% of SDH excitatory interneurons express SP (Gutierrez-Mecinas et al., 2017) and that ablation of a broader population of excitatory interneurons (those that express somatostatin) results in a dramatic reduction in these reflexes (Duan et al., 2014). Our final aim was therefore to test the effect of silencing spinal Tac1-expressing cells on nocifensive reflexes.

## Experimental procedures

### Animals

All experiments were approved by the Ethical Review Process Applications Panel of the University of Glasgow, and were performed in accordance with the European Community directive 86/609/EC and the UK Animals (Scientific Procedures) Act 1986.

We used two genetically modified mouse lines during the course of this study. One of these was the Tac1^Cre^ line (Harris et al., 2014), in which Cre recombinase was knocked into the *Tac1* locus (Tac1-IRES2-Cre-D; The Jackson Laboratory, Bar Harbor, ME, USA; Stock number 021877) and the other was the BAC transgenic Tg(Grp-EGFP)DV197Gsat in which eGFP is expressed under control of the GRP promoter (Gong et al., 2003; Gutierrez-Mecinas et al., 2014; Solorzano et al., 2015). We have recently shown that virtually all eGFP-positive cells in this line possess GRP mRNA, although the mRNA is found in many cells that lack eGFP (Dickie et al., 2019; Bell et al., 2020).

### Analysis of excitatory synaptic input to SP cells

Three Tac1^Cre^ mice (either sex, 19–23 g) were anaesthetised with isoflurane and received intraspinal injections of one of the AAV Brainbow vectors (Cai et al., 2013), which codes for Cre-dependent, membrane-targeted teal fluorescent protein (TFP) and mCherry (AAV9-EF1a-BbChT, referred to as AAV-BB2). Details of all viral injections are provided in Table 1. Injections were made into the dorsal horn of the L3 segment on one side, as described previously (Dickie et al., 2019). Animals received peri-operative analgesia (buprenorphine 0.3 mg/kg and carprofen 5 mg/kg). Following a recovery period of 14 days, the animals were re-anaesthetised with pentobarbitone (20 mg) and perfused through the left ventricle with fixative that contained 4% freshly depolymerised formaldehyde. Spinal cord tissue was removed and postfixed at 4  °C for 2 h. Tissue from these animals was used to analyse synaptic input to SP cells from axons of three classes of excitatory interneuron: those expressing neurotensin, NKB or SP. Parasagittal 60 μm thick sections were cut from the injected side of the L3 segment and processed for immunohistochemistry, as described previously (Gutierrez-Mecinas et al., 2016a, Dickie et al., 2019). Initially, they were reacted with primary antibodies against TFP and Homer (see Table 2), and secondary antibodies labelled with biotin and Alexa488, respectively, before being incubated in Avidin-Pacific Blue to reveal TFP. Sections were then examined with a confocal microscope (Zeiss LSM 710) to identify those containing suitable TFP-positive cells that were sufficiently separated to allow subsequent reconstruction. These sections were then immunoreacted to reveal VGLUT2 and one of the following neuropeptides: NKB, neurotensin or SP, and these were detected with secondary antibodies labelled with Alexa647 and Rhodamine Red, respectively. All secondary antibodies were species-specific, raised in donkey and obtained from Jackson ImmunoResearch, West Grove, PA, USA). They were diluted 1:500 (Alexa488, Alexa647, biotin) or 1:100 (Rhodamine Red). Avidin-Pacific Blue (Life Technologies, Paisley, UK) was diluted 1:1000. All antibodies were diluted in PBS that contained 5% donkey serum, 0.3 M NaCl and 0.3% Triton X-100. Sections were mounted in anti-fade medium and stored at –20 °C.

**Table 1:** AAV vectors

						Details of injection	
	Serotype	Promoter	Construct	Source	Catalogue number	Number of GCs	Volume
AAV.flex.eGFP	AAV1	CAG	eGFP	VVF Zurich	v158-8	2.6 × 10^8^	300 nl
AAV-EF1a-BbChT(AAV-BB2)	AAV9	hEF1a	mTFP, mCherry	Addgene	45,186	7.4 × 10^6^	500 nl
AAV.flex.TeLC	AAV1	hEF1a	TeLC-Flag-tag	H. Wildner		2.03 × 10^8^	300 nl
AAV.flex.TeLC-eGFP	AAV2	hSyn1	TeLC-2A, eGFP*	VVF Zurich	v322-2	2.01 × 10^8^	300 nl

* The AAV.flex.TeLC-eGFP codes for TeLC and eGFP as separate proteins. Injection volumes are listed for each individual injection. GC: gene copies. mTFP: membrane-targeted teal fluorescent protein.

**Table 2:** Antibodies used in this study

Antibody	Species	Catalogue no	Dilution	Source
TFP	Rat	EMU103	1:500	Kerafast
Homer	Goat		1:1000	M Watanabe
VGLUT2	Guinea pig	AB2251	1:5000	Millipore
NKB	Rabbit		1:1000	P Ciofi
Neurotensin	Rabbit	20,072	1:5000	Immunostar
SP	Rabbit	T-4107	1:1000	Peninsula
eGFP	Chicken	ab13970	1:1000	Abcam
CTb	Goat	703	1:1000	List Biological

A similar approach was used to investigate inputs from GRP-eGFP cells to SP cells. In this case, 3 GRP::eGFP;Tac1^Cre^ mice (either sex, 19–21 g) received injections of AAV-BB2. These were performed as described above, except that in this case injections were made into the dorsal horn of both L3 and L5 segments on one side. As before, the mice survived for 14 days and were then fixed by vascular perfusion. Parasagittal sections were cut from the injected segments and reacted with primary antibodies against TFP, eGFP, Homer and VGLUT2 (Table 2), followed by secondary antibodies conjugated to biotin, Alexa488, Rhodamine Red and Alexa647, respectively. Sections were incubated with Avidin-Pacific Blue, mounted and stored, as described above.

For each type of input, 3 TFP-labelled cells were selected from each of 3 animals before the staining for axonal markers was visualised. Cells were selected based on the completeness of dendritic labelling and their separation from nearby neurons. The selected cells were scanned on the confocal microscope through a 63× oil-immersion lens (numerical aperture 1.4), with the aperture set to less than 1 Airy unit. Z-series (0.3 μm z-separation) were obtained from as much of the dendritic tree as was visible in the section. The resulting scans were analysed with Neurolucida for Confocal software (MBF Bioscience, Williston, VT, USA). The TFP and Homer channels were initially viewed. The cell bodies and dendritic trees were drawn, and the locations of all Homer puncta associated with the cell body or with dendritic shafts or spines were plotted. The VGLUT2 channel was then viewed and we noted whether or not a VGLUT2-immunoreactive bouton was apposed to each Homer punctum. Finally, the remaining channel (corresponding to NKB, neurotensin, SP or GRP-eGFP) was revealed and the presence or absence of staining was noted for each of the VGLUT2 boutons that contacted a Homer punctum on the selected cell. To determine the frequency of all boutons arising from excitatory interneurons that were positive for each of these neurochemical markers, we sampled from those VGLUT2-immunoreactive boutons in the vicinity of the TFP-labelled cell. A 4 × 4 μm grid was applied within a box drawn to include the entire dendritic tree of the cell. Only the VGLUT2 channel was viewed initially and in each successive grid square, the VGLUT2-immunoreactive bouton nearest the bottom right of the square was selected. The presence or absence of NKB, neurotensin, SP or GRP-eGFP was then recorded for each of these selected VGLUT2-immunoreactive boutons. Although unmyelinated primary afferents express VGLUT2 (Brumovsky et al., 2007), the expression level of the protein is generally very low in their central terminals, which allows these to be distinguished from boutons belonging to excitatory dorsal horn neurons, which show strong VGLUT2-immunoreactivity (Todd et al., 2003).

### SP-expressing projection neurons

To determine the proportion of lamina I ALT cells that express SP, we combined retrograde tracing with cholera toxin B subunit (CTb) from the lateral parabrachial area (LPb), which labels the vast majority of these cells (Cameron et al., 2015), with intraspinal injection of AAV.flex.eGFP in Tac1^Cre^ mice to reveal neurons that express SP (Gutierrez-Mecinas et al., 2018, Dickie et al., 2019). Two Tac1^Cre^ mice (male, 22–25 g) were anaesthetised with isoflurane and placed in a stereotaxic frame. They received a single injection of 300 nl 1% CTb targeted on the left LPb, as described previously (Cameron et al., 2015). Immediately following this, the T12 and L1 vertebrae were clamped, and intraspinal injections of AAV.flex.eGFP (300 nl) were performed as described previously (Huang et al., 2018). Each mouse received an injection into the dorsal horn in the L5 segments on the right side, and in one case there was an additional injection in the L3 segment on this side. Perioperative analgesia (buprenorphine 0.3 mg/kg and carprofen 5 mg/kg) was provided and the animals were allowed to survive for 3 days. They were then anaesthetised with pentobarbitone and perfused with fixative, as described above. Brain and lumbar spinal cord segments were removed and post-fixed for 2 h. The brain was cut into 100 μm thick coronal sections, which were reacted for CTb with an immunoperoxidase method (Cameron et al., 2015). Spinal cord segments were cut into 60 μm thick transverse sections, and these were reacted with antibodies against CTb and eGFP, and subsequently in secondary antibodies conjugated to Alexa488, Rhodamine Red or biotin. Biotinylated secondary antibodies were detected with Avidin-Pacific Blue.

Sections through the spinal injection sites were scanned with the confocal microscope through a 20× dry lens (numerical aperture 0.8) to generate z-stacks (1 μm z-separation) of the superficial dorsal horn through the full thickness of the section. These were analysed with Neurolucida for Confocal software. Initially, the region of dorsal horn corresponding to the injection site was defined, by examining the mediolateral distribution of eGFP-positive cells. All CTb-labelled lamina I neurons within this region were then identified, and the presence or absence of eGFP was noted for each cell.

### Silencing of dorsal horn SP cells

Experiments to test the effect of silencing SP cells were performed on 23 Tac1^Cre^ mice of either sex (15–26 g, 12 female). Intraspinal injections were performed as described above, and all animals received three injections targeted on the L3, L4 and L5 segments on the right side (Huang et al., 2018). Injections consisted of AAV containing a Cre-dependent construct coding for either tetanus toxin light chain (TeLC) or eGFP (Table 1), and animals were randomly assigned to experimental (TeLC, *n* = 12, 6 female) or control (eGFP, *n* = 11, 6 female) groups. In the initial 8 TeLC experiments, we used a vector that coded for TeLC and Flag-tag (AAV.flex.TeLC-Flag). However, we were unable to visualise the Flag-tag by immunohistochemistry, and for the last 4 experiments we used a vector that coded for both TeLC and eGFP (as separate proteins).

Behavioural tests were performed before and after spinal injections. All tests and analyses were performed blind to the treatment type. Baseline tests (von Frey, Hargreaves, cold, Rotarod) were performed 4–5 days before spinal injection, and these tests, together with a test for pruritogen-evoked itch, were repeated between 8 and 23 days after surgery. Mechanical sensitivity was tested with von Frey hairs. Animals were acclimatised for 1 h in a cage with a wire mesh floor and then tested by application of filaments with logarithmically incremental stiffness (starting at 0.4 g) to the glabrous skin of the hindpaw. Each filament was applied for 5 s, and the presence or absence of a withdrawal response was noted. Depending on the response, the filament with the next incremental stiffness was tested. Testing continued until a series of six filaments had been applied from the point when the response threshold was first crossed. The 50% withdrawal threshold was determined by the up-down method (Chaplan et al., 1994). Thermal (heat) sensitivity was tested with a Hargreaves apparatus (IITC, Woodland Hills, CA, USA). Animals were acclimatised for 1 hour on a glass plate warmed to 25 °C and a radiant heat source was then targeted at each hindpaw 5 times with a 10 min interval between tests. The time taken to lift the paw was measured. To test cold sensitivity, mice were placed in a plastic enclosure on a 3 mm-thick glass plate that was at room temperature, and allowed to acclimatise for at least 45 min. A dry ice pellet of ∼1 cm diameter was applied to the underside of the glass directly below the hindpaw to be tested, and the time taken to withdraw the paw was recorded (Brenner et al., 2015). Care was taken to ensure that the plantar surface of the hindpaw was in direct contact with the glass prior to testing. Testing of ipsilateral and contralateral paws was alternated with at least 3 min interval between consecutive tests. Each hindpaw was tested 5 times, and the average withdrawal latency calculated. Motor co-ordination was tested with a Rotarod (IITC), with the rod programmed to accelerate from 4 to 40 rpm over 5 min exactly as described previously (Huang et al., 2018). Pruritogen-evoked itch was tested by intradermal injection of chloroquine into the calf (LaMotte et al., 2011, Kardon et al., 2014, Huang et al., 2018). Animals were acclimatised for 2 h in a plastic observation chamber that was surrounded by mirrors, to provide an unobstructed view of the hindlimb. Chloroquine (100 μg dissolved in 10 μl of PBS) was injected into the right calf (which had been shaved at least 24 h previously), and in each case success of the injection was confirmed by the presence of a bleb. Mice were video-recorded for 30 mins before and after the chloroquine injection and the time spent biting the injection site was analysed later off-line.

### Characterisation of antibodies

The sources and dilutions of primary antibodies used in the study are listed in Table 2. The TFP and eGFP antibodies were raised against full-length proteins and specificity is shown by the lack of staining in regions away from the injection sites. The affinity-purified Homer antibody was raised against amino acids 1–175 of mouse Homer 1 and detects a band at 43–45 kDa in immunoblots of mouse brain extracts. Since the first 120 amino acids are highly conserved between Homer 1, 2 and 3 the antibody is likely to detect all forms of Homer. We have shown that punctate staining with this antibody is associated with glutamatergic boutons in the spinal dorsal horn (Gutierrez-Mecinas et al., 2016b). The VGLUT2 antibody was raised against a peptide corresponding to amino acids 565–582 of rat VGLUT2 and stains identical structures to a well-characterised rabbit VGLUT2 antibody (Todd et al., 2003). The NKB antibody was raised against a 40 amino-acid sequence from the rat preprotachykinin B (the precursor to NKB), and immunostaining was abolished by pre-incubation with the immunizing peptide at 10^−5^ M (Ciofi et al., 2006). The neurotensin antibody was raised against synthetic human neurotensin conjugated to bovine thyroglobulin. Immunostaining is abolished by pre-incubation with 10 μg neurotensin per ml of diluted antibody (manufacturer's specification). The SP antibody was raised against the sequence H-Arg-Pro-Lys-Pro-Gln-Gln-Phe-Phe-Gly-Leu-Met-NH_2_. It detects SP and peptides containing at least the last 6 amino acids (Gln-Phe-Phe-Gly-Leu-Met-NH_2_), but does not cross-react with either neurokinin A or NKB (manufacturer's specification). The CTb antibody was raised against the purified protein, and specificity is demonstrated by the lack of staining in regions that did not contain injected or transported tracer.

### Statistics

For the analysis of synaptic inputs to the SP cells from different neurochemical populations of excitatory interneurons, comparisons between the proportion of VGLUT2 boutons synapsing on the cell and those in the vicinity of the cell that were positive for each marker were made using Wilcoxon matched pairs tests. Mann-Whitney U test was used to compare the proportion of input arising from VGLUT2-positive boutons to SP and GRP-eGFP cells.

For the analysis of behavioural data in the studies involving synaptic silencing we used 2-way ANOVA (itch and Rotarod) or 3-way ANOVA (nocifensive reflex tests) followed by Tukey's multiple comparisons test *post hoc* when ANOVA showed significant differences in the main effects or interactions (*p* < 0.05).

## Results

### Brainbow labelling of SP cells and analysis of synaptic inputs

**Fig 1:**
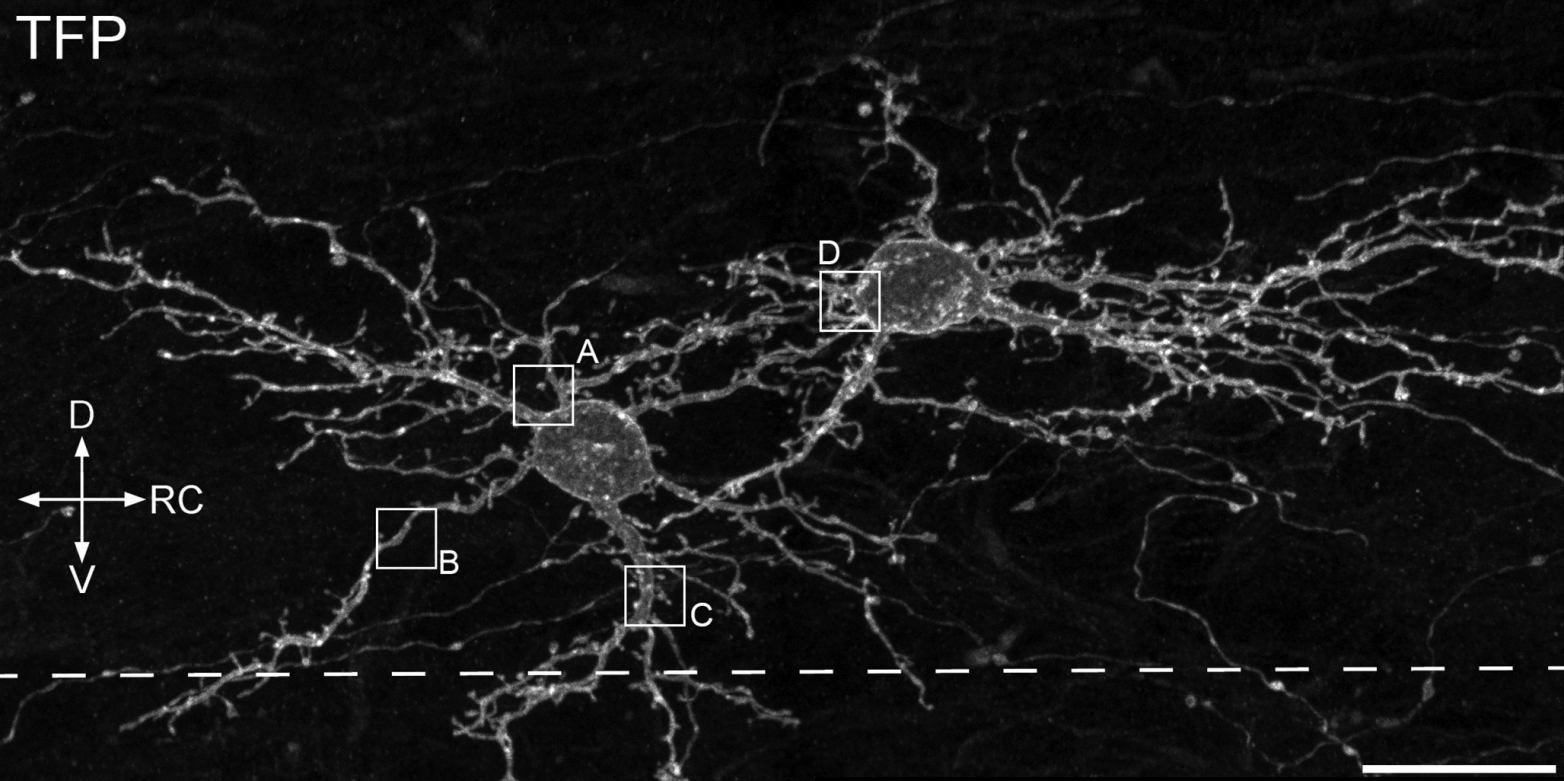
Substance P-expressing cells in lamina II that were labelled with the Brainbow technique, following injection of AAV-BB2 into the lumbar dorsal horn of a Tac1^Cre^;GRP::eGFP mouse. A sagittal section stained to reveal teal fluorescent protein (TFP). Two labelled cells are visible, and both show the characteristic morphological features of radial cells, with round cell bodies, several primary dendrites and a compact dendritic tree. The image shows a maximum intensity projection of 48 confocal optical sections at 0.3 μm z-spacing. Boxes indicate the regions illustrated in Fig. 2. The dashed line represents the approximate position of the lamina II-III border. D, dorsal; RC, rostrocaudal; V, ventral. Scale bar = 20 μm.

The amount of Brainbow virus injected in these experiments was intentionally kept very low, such that the number of labelled cells was limited, and the native fluorescence within these cells was barely detectable. Immunostaining for TFP resulted in the appearance of scattered immunoreactive cells, with a laminar distribution that was similar to what we had previously observed (Dickie et al., 2019). The cells were largely restricted to lamina II, and typically resembled the radial cells described by Grudt and Perl (2002) (Fig. 1). They generally had several primary dendrites that gave rise to highly branched, compact dendritic trees that did not extend far from the soma. The dendrites had a moderate density of spines with 17.7 spines (±5.5 SD) per 100 μm of dendrite. Homer puncta were associated with both dendritic shafts and spines. In a survey of 528 spines on 10 different SP cells, we identified Homer puncta at 497 (94.2%) of these, consistent with the view that dendritic spines represent a major site of excitatory synaptic input.

**Fig 2:**
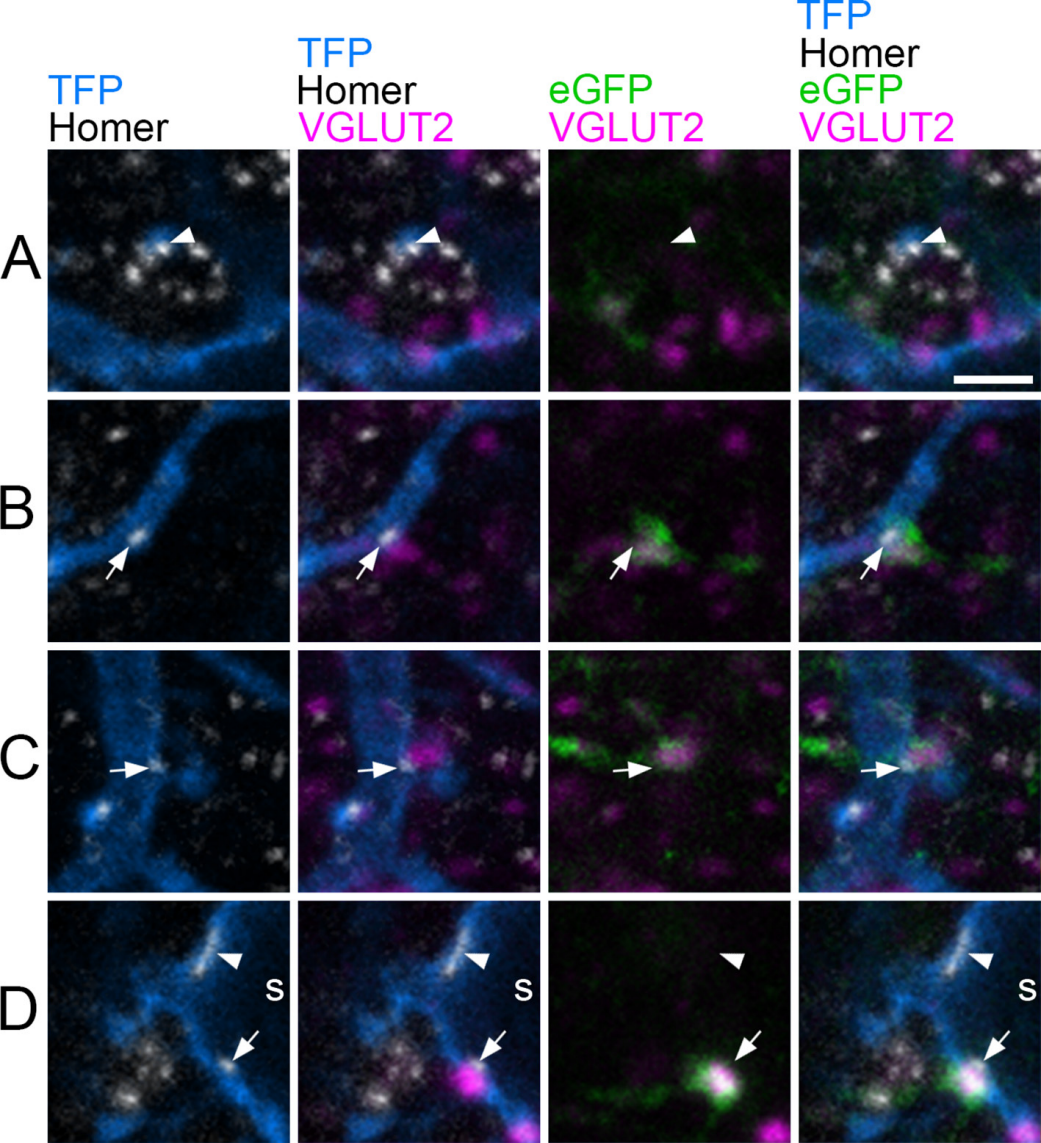
Excitatory synapses on the Brainbow labelled substance P cells that are illustrated in Fig. 1, revealed by immunostaining for Homer. **(A–D)** each row shows a field corresponding to the boxes in Fig. 1. The first column shows teal fluorescent protein (TFP, blue), and Homer (grey). Arrows and arrowheads point to Homer puncta (which represent excitatory synapses) on a dendritic spine **(A)**, on dendritic shafts **(B, C)** and on the soma (s) of one of the cells **(D)**. The second column also shows VGLUT2 (magenta), which is present at some of the sites where Homer puncta are associated with the labelled cells (those shown with arrows in **(B–D)**). Other Homer puncta (those shown with arrowheads in **(A, D)**) lack detectable VGLUT2 staining. The ring of Homer puncta seen near the centre of the field in A is likely to be formed by excitatory synapses associated with the central (VGLUT2-negative) bouton of a synaptic glomerulus. In the 3rd column, VGLUT2 is shown together with eGFP (green), which labels the GRP-eGFP cells and their processes. The three profiles marked with arrows in B–D are all eGFP-positive, indicating that they are axonal boutons that originate from GRP-eGFP cells. The fourth column shows merged images with all four types of immunostaining. All images were obtained from single confocal optical sections. Scale bar (for all parts) = 2 μm.

**Fig 3:**
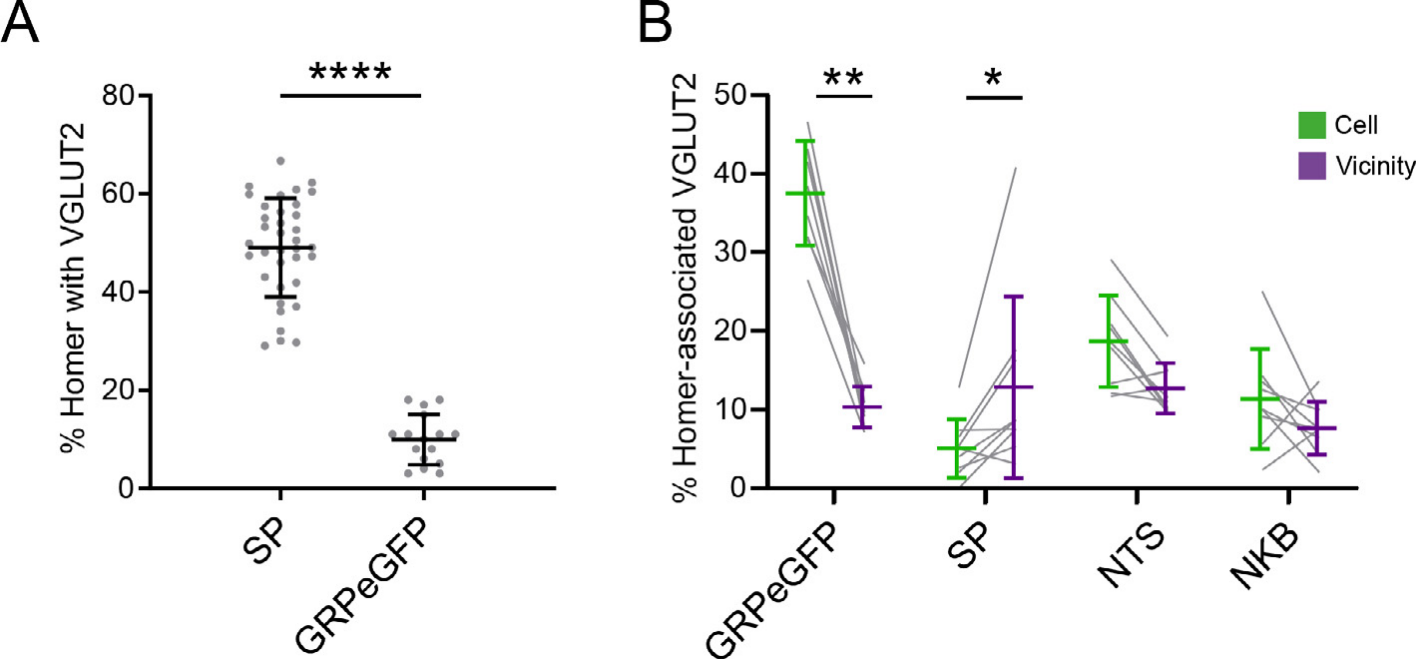
Association of VGLUT2-immunoreactive boutons with substance P (SP) cells. **(A)** Scatter plot showing the percentage of Homer puncta on the 36 SP cells analysed in this study that were associated with VGLUT2-immunoreactive boutons. For comparison, corresponding results are also shown for 16 GRP-eGFP cells that were analysed as part of a separate study (Bell et al (2020)). The difference between these two populations is highly significant (*p* < 0.0001; Mann-Whitney *U* test). **(B)** comparison of the percentage of VGLUT2-immunoreactive boutons associated with Homer puncta on the SP cells that were positive for 4 different markers (9 cells examined in each case), compared with the percentage of VGLUT2 boutons in the vicinity of each cell that were positive for the corresponding marker. The first column shows VGLUT2 boutons that were eGFP-positive in Tac1^Cre^;GRP::eGFP mice. The remaining columns show VGLUT2 boutons in Tac1^Cre^ mice that were immunoreactive for substance P (SP), neurotensin (NTS) or neurokinin B (NKB), respectively. The differences for eGFP-positive boutons in the Tac1^Cre^; GRP::eGFP mice and for SP boutons in the Tac1^Cre^ mice were both significant (p = 0.004 and p = 0.018, respectively, Wilcoxon matched pairs tests).

Thirty-six TFP-labelled cells were reconstructed in full (9 each from 3 Tac1^Cre^ mice and 3 each from 3 Tac1^Cre^;GRP::eGFP mice) and details of the dendritic lengths and the total number of Homer puncta associated with each cell are given in Table 3. Overall, 51% (±12%, SD) of the Homer puncta were detected on dendritic spines (Fig. 2A), and most of the remainder were on dendritic shafts (Fig. 2B, C), with a few being present on cell bodies (Fig. 2D). In order to distinguish boutons with strong or medium VGLUT2 immunoreactivity (most of which are likely to originate from local interneurons) from those with very low levels of VGLUT2 (which are likely to correspond to primary afferents) we initially assessed the level of VGLUT2 in putative synaptic glomeruli. These can be identified in lamina II as clusters of Homer puncta that surround a central region that either lacks VGLUT2 or shows extremely weak immunoreactivity (Fig. 2A). Any boutons showing stronger VGLUT2 immunoreactivity than these were counted as VGLUT2-positive. The proportion of Homer puncta that were apposed to VGLUT2-immunoreactive boutons ranged from 29.7% to 66.7% (mean 49%) (Fig 2, Fig 3A). We have recently characterised the excitatory synaptic input to GRP-eGFP cells and this figure is significantly higher than seen in that population of cells (*p* < 0.0001, Bell et al., 2020) (Fig. 3A).

**Table 3:** Details of neuron tracing and synaptic quantification

Marker	Mice	Cells	Dendritic length (μm/ cell)	Homer puncta/cell	No. Homer with VGLUT2	On cell		In vicinity of cell		
						No. VGLUT2 with Marker	% VGLUT2 with Marker	Total VGLUT2 boutons	No. VGLUT2 with Marker	% VGLUT2 with Marker
SP	3*	9	659 (419–930)	233 (155–274)	93 (47–148)	6 (0–19)	5.1 (0.0–12.8)	90 (85–97)	11 (3–29)	12.8 (3.1–40.8)
NTS	3*	9	805 (490–1340)	267 (170–385)	125 (58–182)	24 (9–44)	18.7 (11.7–29.1)	114 (100–175)	14 (10–21)	12.7 (9.7–19.3)
NKB	3*	9	600 (360–807)	201 (152–347)	105 (72–168)	12 (2–24)	11.4 (2.3–25)	93 (88–98)	7 (2–12)	7.6 (2.0–13.6)
GRPeGFP	3	9	887 (720–1001)	320 (231–427)	180 (142–252)	66 (41–94)	37.5 (31.7–46.6)	132 (102–231)	9 (8–21)	10.3 (7.8–15.8)

Values are means, with ranges shown in brackets.

* The same 3 Tac1^Cre^ mice were used to generate tissue for the analysis of synaptic input from SP-, neurotensin- (NTS) and NKB-immunoreactive boutons.

Nine SP cells were analysed from 3 Tac1^Cre^;GRP::eGFP mice, and we found that 37.5% of the VGLUT2-immunoreactive boutons apposed to Homer puncta on these cells were GRP-eGFP-positive (Fig 2, Fig 3B, Table 3). For comparison, only 10.3% of the VGLUT2 boutons that were in the vicinity of the cell showed eGFP and this difference is highly significant (*p* = 0.004; Wilcoxon matched pairs).

**Fig 4:**
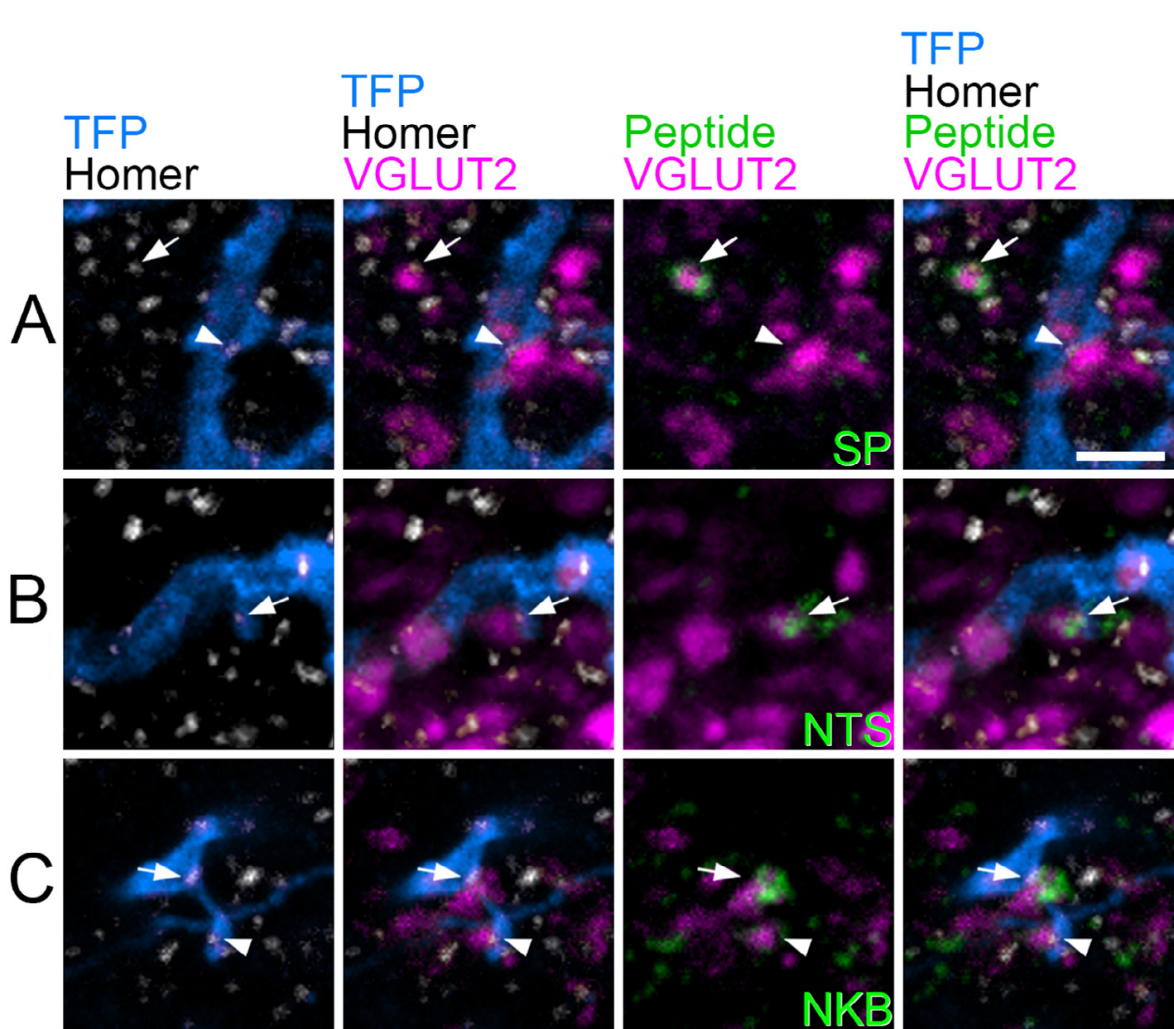
Association of SP cells with axons derived from excitatory interneurons that express different neuropeptides. **(A–C)** Show fields from Tac1^Cre^ mice that had received AAV.BB2 injections and had then been reacted with antibodies against TFP, Homer, VGLUT2 and either SP **(A)**, neurotensin (NTS, **(B)**) or NKB **(C)**. The layout is equivalent to that shown in parts A-D of Fig. 2. **(A)** A TFP-labelled dendrite contains a Homer punctum (arrowhead), which is associated with a VGLUT2 bouton that lacks SP. A nearby SP-positive VGLUT2 bouton is indicated with an arrow. **(B)** A small Homer punctum on a TFP-labelled dendritic spine (arrow) is adjacent to a NTS-positive VGLUT2 bouton. **(C)** A TFP dendrite and attached dendritic spine have two Homer puncta. One of these (arrow) is adjacent to a VGLUT2 bouton with strong NKB-immunoreactivity, while the other (arrowhead) is in contact with a VGLUT2 bouton that shows very weak NKB labelling. Scale bar (A–C) = 2 μm.

We analysed 27 cells in the Tac1^Cre^ mice, 9 of which were from sections that had been reacted with each neuropeptide antibody (SP, neurotensin, NKB) (Figs. 3B, 4, Table 3). Immunoreactivity for SP, neurotensin and NKB was detected in 5.1%, 18.7% and 11.4%, respectively, of the VGLUT2-immunoreactive boutons apposed to Homer puncta on these cells. For comparison, 12.8%, 12.7% and 7.6% of VGLUT2 boutons in the vicinity of the cells were positive for these neuropeptides (Table 3). Wilcoxon matched pairs tests showed that SP was present in a significantly lower proportion of those VGLUT2 boutons that synapsed on SP cells than was the case for nearby VGLUT2 boutons (*p* = 0.018), suggesting that SP boutons tend to avoid other SP cells. In the case of the other two peptides, there was no significant difference (*p* = 0.076 for neurotensin, *p* = 0.17 for NKB), although there was a trend for neurotensin to be more frequently present in boutons synapsing on the SP cells than in nearby VGLUT2 boutons.

### SP expression by lamina I projection neurons

**Fig 5:**
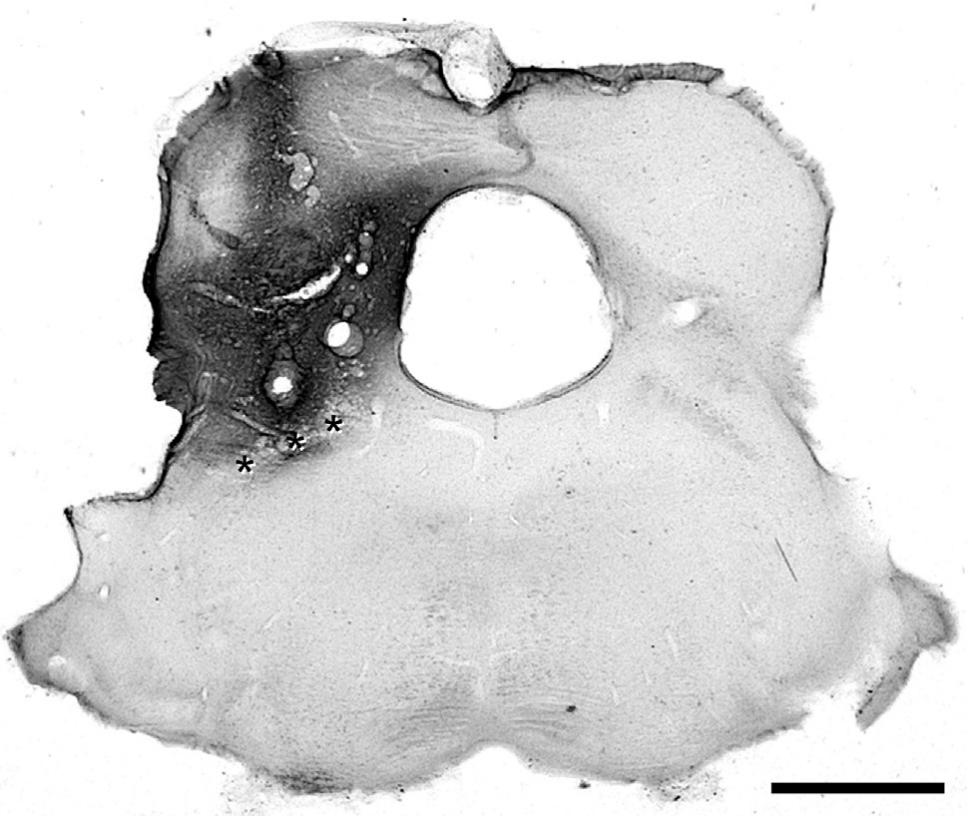
Photomicrograph of cholera toxin B (CTb) injection site. A transverse section through the brainstem from one of the mice that received an injection targeted on the lateral parabrachial area, showing the immunoperoxidase labelling for CTb. The position of the superior cerebellar peduncle is indicated with asterisks. CTb is present throughout the LPb and extends into the caudal part of the periaqueductal grey matter. Scale bar = 1 mm.

**Fig 6:**
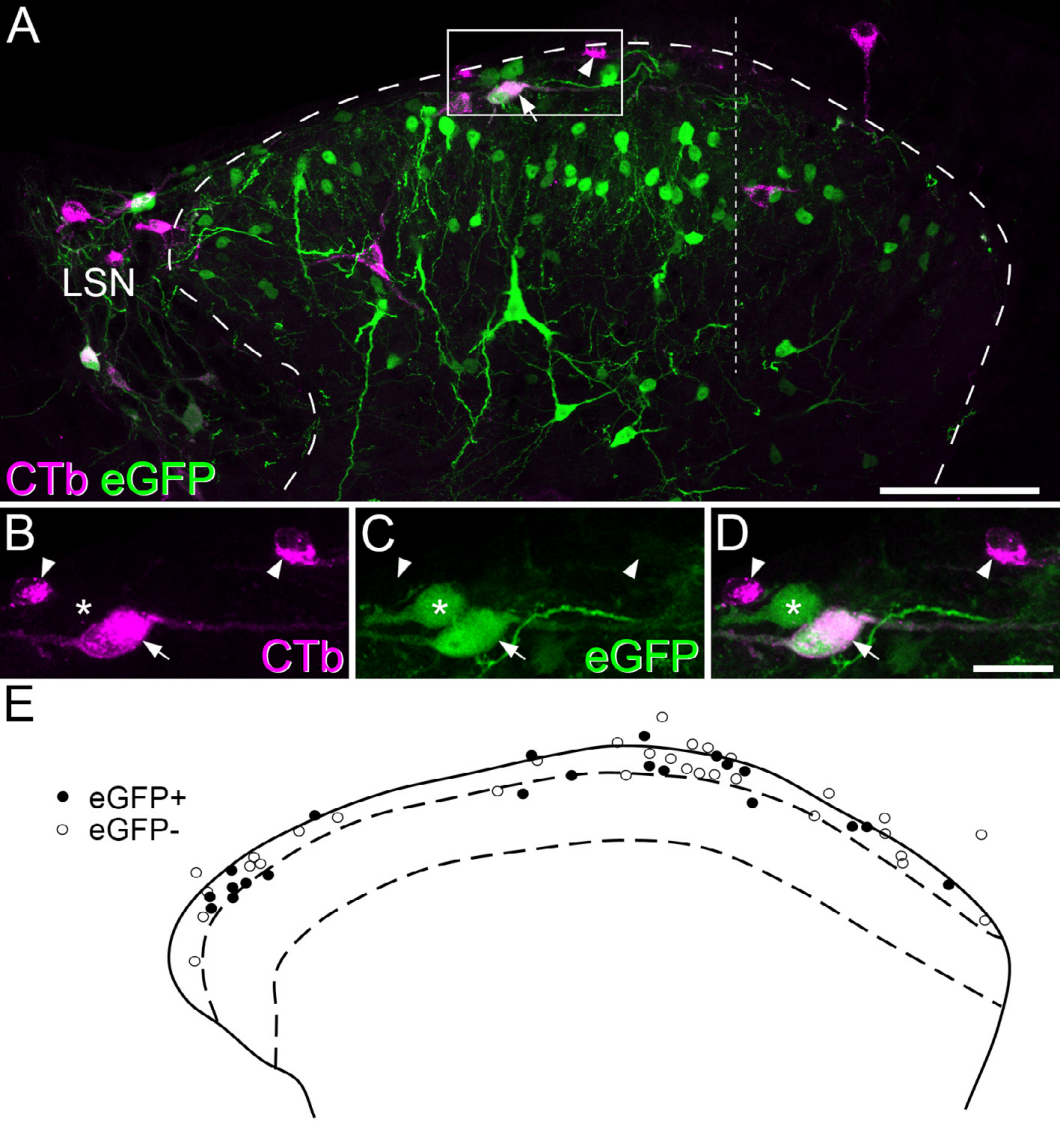
Retrograde labelling from the lateral parabrachial (LPb) area combined with intraspinal injection to label substance P cells in a Tac1^Cre^ mouse. Cholera toxin B (CTb) was injected into the LPb on one side and AAV.flex.eGFP into the L5 dorsal horn on the contralateral side. **(A)** A transverse section through the L5 segment (corresponding to the intraspinal injection site) that has been immunostained to reveal CTb (magenta) and eGFP (green). Numerous eGFP-positive cells are present throughout the superficial laminae and scattered cells are present in the deeper part. CTb-positive cells, which have been retrogradely labelled from the LPb, are most numerous in lamina I and the lateral spinal nucleus (LSN). Some of these are also positive for eGFP (one shown with an arrow) and others lack eGFP (one shown with an arrowhead). **(B–D)** A detail from the region shown in the box in **(A**). A double-labelled cell is indicated with an arrow, two CTb-positive/eGFP-negative cells with arrowheads and a CTb-negative/eGFP-positive cell with an asterisk. The thicker dashed line in **(A)** outlines the dorsal horn, and the thinner dashed lines indicates the medial edge of the AAV.flex.eGFP injection site. **(E)** Positions of retrogradely-labelled superficial dorsal horn neurons that were eGFP+ (filled symbols) and eGFP- (open symbols) in one of the two experiments, plotted onto an outline of the grey matter. The dashed lines represent the approximate positions for the borders of lamina II. Images in **(A)** and **(B–D)** were from 10 and 4 optical sections at 1 μm z-spacing, respectively. Scale bars: A = 100 μm; B–D = 25 μm.

The CTb injections in the two Tac1^Cre^ mice completely filled the parabrachial area in each case, with some extension into nearby regions, including the caudal part of the periaqueductal grey matter (Fig. 5). Numerous retrogradely-labelled (CTb-immunoreactive) cells were seen in lamina I and the LSN on the contralateral side, and there were also scattered cells in deeper laminae and on the side ipsilateral to the injection site (Fig. 6A). The AAV.flex.eGFP injections partially filled the dorsal horn in the L5 segment in each case, and the L3 segment in one animal. Altogether 109 CTb-labelled cells were identified in or close to lamina I and within the zone occupied by the viral injection site from the 2 mice (52 and 57 cells from each mouse). Overall, 42.5% of these cells (21/52, and 25/57; 40.4% and 43.9%) were eGFP-positive, and examples are shown in Fig. 6A–D. The eGFP-positive and eGFP-negative CTb-labelled cells were evenly distributed across the mediolateral axis (Fig. 6E).

### Effect of silencing dorsal horn SP cells

**Fig 7:**
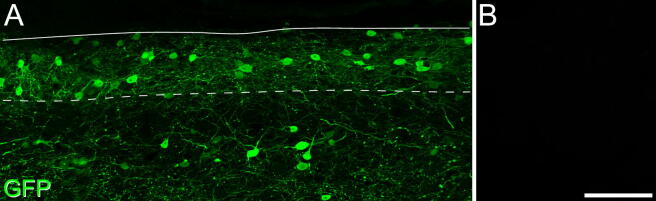
The distribution of enhanced green fluorescent protein (eGFP) seen following intraspinal injection with AAV.flex.TeLC-eGFP. **(A)** Sagittal section through part of the ipsilateral L4 segment from one of the mice that received an intraspinal injection of the vector coding for both TeLC and eGFP. Labelled cells are present throughout the dorsal horn, but are concentrated in the superficial part. Upper and lower lines indicate the dorsal border of the grey matter and the approximate position of the lamina II/III border, respectively. **(B)** A scan for eGFP through the ipsilateral L4 dorsal root ganglion. No eGFP labelling is visible, indicating the lack of labelled primary afferents following this injection strategy. Images in A and B are projections of 3 and 7 confocal images at 3 μm z-separation, respectively. Scale bar = 100 μm.

As mentioned above, we could not detect the Flag-tag in mice that received injections of AAV.flex.TeLC-Flag, and we therefore used AAV.flex.TeLC-eGFP for the last 4 silencing experiments. In each of these cases, we were able to identify the injection site by the presence of eGFP, and this showed the expected pattern of labelling in the ipsilateral dorsal horn, with numerous labelled eGFP-positive cells in the superficial dorsal horn, especially lamina II, and scattered cells in deeper laminae (Fig. 7A). No eGFP-positive cells were detected in the ipsilateral L3-L5 dorsal root ganglia in any of these animals (Fig. 7B).

**Fig 8:**
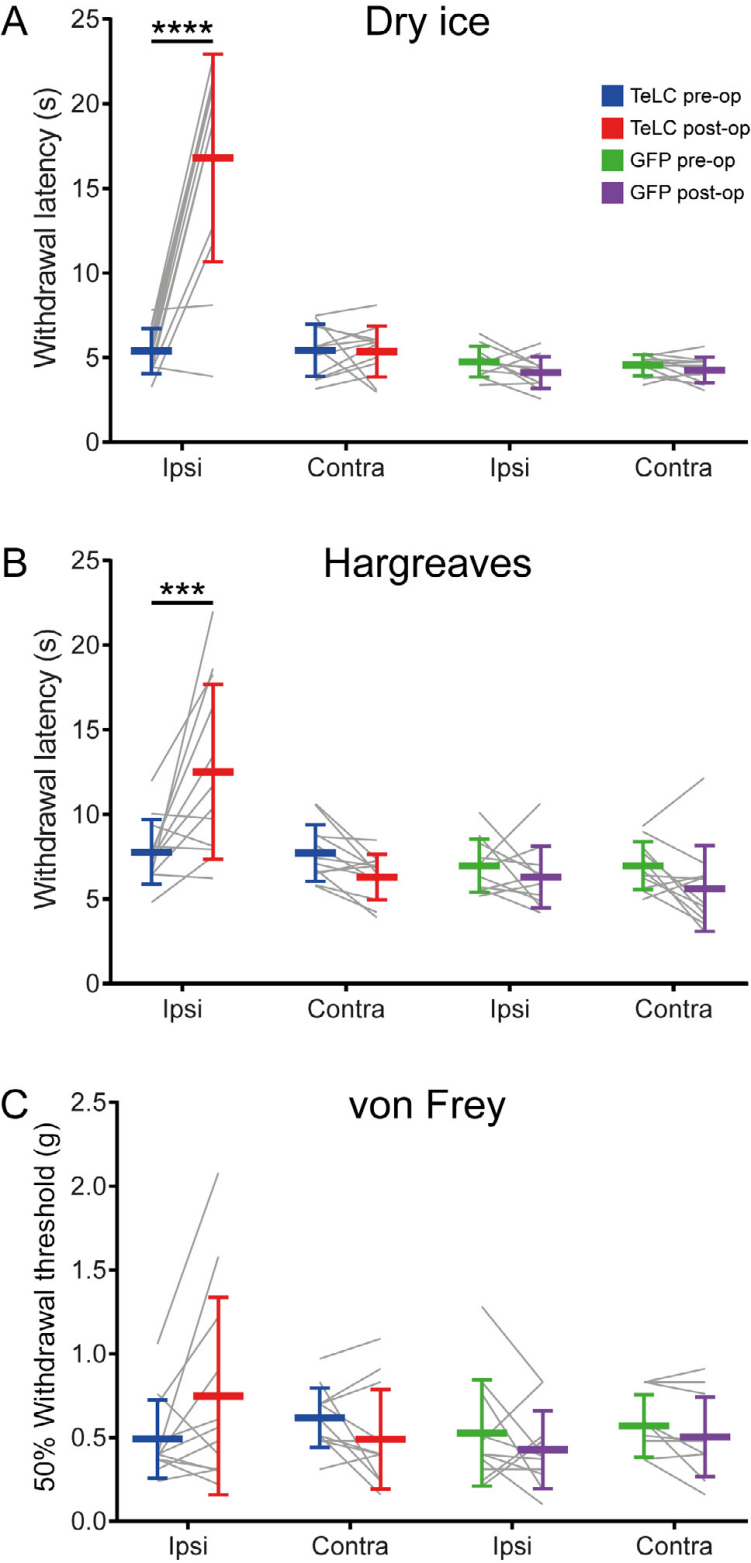
Nocifensive reflex tests for Tac1^Cre^ mice that received intraspinal injections of AAVs coding for either TeLC or eGFP. **(A–C)** Results for the dry ice, Hargreaves and von Frey tests, respectively. For the cold (Dry ice) and heat (Hargreaves) tests, the mice that received TeLC had significantly increased withdrawal latency for the ipsilateral hindlimb when comparing their pre- and post-operative values (*p* < 0.0001 and <0.001, respectively, 3-way ANOVA followed by Tukey's multiple comparisons test). No significant differences were found for the ipsilateral von Frey test in the TeLC-injected mice, for any of the contralateral tests in these mice or for either ipsilateral or contralateral tests in the eGFP-injected mice. Plots show mean and standard deviation. Individual paired values for each mouse are shown with grey lines.

For mice that received injections of AAV.flex.eGFP there was no significant difference in pre-operative and post-operative behaviours in response to dry ice, radiant heat or application of von Frey hairs (Fig. 8). Mice that received injections of AAV coding for TeLC showed a significant increase in latency for withdrawal in both the dry ice and Hargreaves tests (*p* < 0.0001, *p* = 0.0008, respectively; Fig. 8A, B) but no significant change in response to von Frey hairs (Fig. 8C). No changes were seen on the contralateral side in any of the animals.

**Fig 9:**
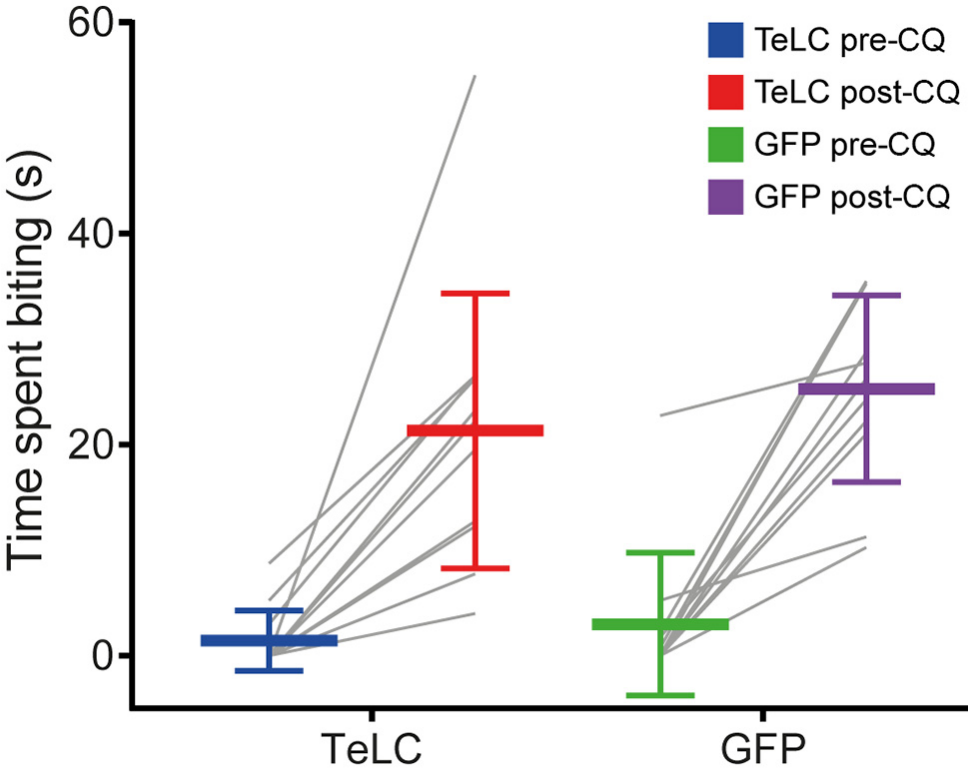
Itch tests for Tac1^Cre^ mice that received intraspinal injections of AAVs coding for either TeLC or eGFP. The cumulative time spent biting within 30 min before and after intradermal injection of chloroquine into the calf ipsilateral to the intraspinal injection site are shown. Although mice showed considerably increased time spent biting after the chloroquine injection, this did not differ between the two groups of mice. Plots show mean and standard deviation. Individual paired values are shown with grey lines.

Intradermal injection of chloroquine resulted in a significant increase in the time spent biting (*p* < 0.0001), but there was no difference in the response between the mice that had received intraspinal injections of TeLC or eGFP (Fig. 9). There were no significant differences in the maximum rpm tolerated in the Rotarod when comparing treatment groups or pre- vs post-operative tests (data not shown).

## Discussion

Our main findings are: (1) that approximately half of the Homer puncta on the SP cells in lamina II are associated with VGLUT2-immunoreactive boutons, which originate mainly from local interneurons, (2) that axons of GRP-eGFP cells are significantly over-represented among these boutons, (3) that around 40% of lamina I spinoparabrachial neurons express Cre in adult Tac1^Cre^ mice, and (4) that synaptic silencing of dorsal horn SP neurons causes a significant reduction in thermal nocifensive reflexes.

### Synaptic input to SP neurons

We adapted the viral Brainbow method (Cai et al., 2013, Dickie et al., 2019), by using only one of the 2 Brainbow AAVs and injecting a small amount of the virus. This resulted in very sparse labelling, and following immunohistochemical detection of one of the fluorescent proteins (TFP) individual cells could be readily identified and their processes followed, allowing more or less complete reconstruction of SP cells and their dendritic trees. Although AAV-BB2 also codes for mCherry, the expression level was so low that it was effectively undetectable, and did not interfere with the immunolabelling that was revealed with Rhodamine Red-labelled secondary antibodies.

As reported previously (Dickie et al., 2019), the SP interneurons in lamina II resembled radial cells described by Grudt and Perl (2002). Our immunohistochemical findings suggest that around half of their excitatory synaptic input originates from boutons with moderate-high levels of VGLUT2, and these are likely be derived mainly from local excitatory interneurons (Todd et al., 2003). We have previously shown that there is minimal overlap between the GRP-eGFP subset of excitatory interneurons and those that express neurotensin, NKB or SP (Gutierrez-Mecinas et al., 2014, Gutierrez-Mecinas et al., 2016a, Dickie et al., 2019 Bell et al., 2020). Our analysis therefore suggests that between them these four populations account for nearly 75% of the VGLUT2-positive synaptic input. The remaining VGLUT2-positive boutons could be derived from other populations of SDH excitatory interneurons, such as those that express CCK or NPFF (Häring et al., 2018, Gutierrez-Mecinas et al., 2019a, Gutierrez-Mecinas et al., 2019b), from local collaterals of ALT projection neurons (Szucs et al., 2010), or potentially from axons that descend from the brainstem. The remaining 50% of the Homer puncta (presumed glutamatergic synapses) on the SP cells were not associated with VGLUT2-immunoreactive boutons, and the presynaptic axons at these synapses are likely to originate mainly from unmyelinated primary afferents, which generally lack detectable VGLUT2 (Todd et al., 2003). Consistent with this interpretation, radial cells have been shown to receive monosynaptic input from both C and Aδ afferents (Grudt and Perl, 2002, Yasaka et al., 2007). There may also be a minor contribution from corticospinal tract axons, which terminate sparsely in lamina II (Cheema et al., 1984, Liu et al., 2018), and which express VGLUT1 but not VGLUT2 (Abraira et al., 2017).

We found that GRP-eGFP axons consistently accounted for around one third of the VGLUT2-positive boutons that innervated the SP cells, but for only around 10% of the nearby VGLUT2 boutons. We have previously shown that the SP and GRP-eGFP cells in lamina II largely correspond to two of the classes defined by Grudt and Perl (2002): radial and transient central cells, respectively (Dickie et al., 2019). The present results provide evidence that the SP cells are relatively homogeneous in terms of their synaptic inputs, and suggest that they are preferentially innervated by transient central (GRP-eGFP) cells. We had already shown that these two populations differ in terms of their morphology, electrophysiological properties, receptor expression patterns and propriospinal projections (Gutierrez-Mecinas et al., 2018, Dickie et al., 2019). Here we provide evidence that there are also major differences in the proportion of their excitatory synaptic input that originates from primary afferents or spinal interneurons. Previous studies have identified a circuit involving protein kinase Cγ-expressing neurons in laminae IIi-III, transient central cells, vertical cells and lamina I projection neurons, which is thought to underlie tactile allodynia in neuropathic pain states (Lu and Perl, 2005, Lu et al., 2013). However, little was apparently known about synaptic circuits involving radial cells. Our results suggest an additional pathway, in which transient central cells, which receive strong primary afferent input, target radial cells. It will therefore be important to determine the postsynaptic targets of the radial cells. Little is currently known about the function of radial cells, except that they undergo disinhibition following peripheral nerve injury (Imlach et al., 2016).

Many SP interneurons in lamina II have been shown to express enkephalin (Senba et al., 1988, Ribeiro-da-Silva et al., 1991), and this finding is supported by the high levels of both Tac1 and Penk mRNAs in the Glut10-11 class of Häring et al (2018). Interestingly, Sun et al (2017) provided evidence that GRP-eGFP cells were presynaptic to enkephalin-expressing neurons, and this is consistent with our findings of a high frequency of synaptic input from GRP-eGFP cells to SP neurons in lamina II, many of which are likely to co-express enkephalin.

It should be noted that although the approach that we used can reveal the presence of synapses between different dorsal horn neurons, electrophysiological methods, for example optogenetics, will be required to demonstrate the function of these synapses.

### SP expression by lamina I projection neurons

Early evidence of SP expression by ALT projection neurons was provided by Battaglia and Rustioni (1992), who identified SP-immunoreactivity in retrogradely-labelled lamina I spinothalamic neurons in rats treated with intraspinal colchicine, and Noguchi and Ruda (1992), who demonstrated that many lamina I spinoparabrachial neurons in the rat contained mRNA for preprotachykinin A (PPTA, the precursor for SP). It was subsequently shown that spinoparabrachial axon terminals in the cat were frequently immunoreactive for SP (Blomqvist and Mackerlova, 1995). Here we show that at least 40% of the lamina I neurons that were retrogradely labelled from LPb (and which correspond to the vast majority of ALT cells in this lamina) were Cre-positive in the Tac1^Cre^ mouse. Huang et al (2019) recently reported that 37% of lamina I neurons that expressed the neurokinin 1 receptor (NK1r) were labelled with tdTomato when Tac1^Cre^ mice were crossed with a tdTomato reporter line. Since most lamina I cells with high levels of NK1r are ALT neurons (Todd, 2010), our results are consistent with the suggestion that the NK1r population seen by Huang et al included many projection cells, and show that these cells continue to express Tac1 in adulthood.

We previously found that 19% of spinoparabrachial terminals were SP-immunoreactive (Cameron et al., 2015). There are several possible explanations for the apparent discrepancy between this, and our present finding that ∼40% of spinoparabrachial cells are Tac1-positive. The SP-expressing spinoparabrachial neurons may give rise to fewer boutons than the SP-negative cells, and would therefore be under-represented among spinoparabrachial terminals, or alternatively SP may not be present in all of their axonal boutons. It is also possible that despite expressing the *Tac1* gene, some spinoparabrachial cells may not synthesise SP. We have shown that some inhibitory interneurons in lamina II (which correspond to the Gaba9 population of Häring et al) are Tac1-positive and contain the precursor protein PPTA, but do not have detectable levels of SP in their axonal boutons, possibly due to lack of the enzyme peptidylglycine alpha-amidating monooxygenase, which is required for maturation of certain biologically active peptides (Sathyamurthy et al., 2018, Gutierrez-Mecinas et al., 2019c). Finally, the spinoparabrachial terminals identified by Cameron et al may have included axons originating from cells outside lamina I, and the proportion of these cells that are Tac1-positive could be lower.

### Effect of silencing dorsal horn SP neurons

Huang et al (2019) reported that ablation of spinal “Tac1-lineage” neurons (i.e. those cells that expressed Tac1 or had done so during development) did not significantly alter acute nocifensive reflexes, but did reduce itch behaviour in response to intradermally injected chloroquine. In contrast to this, we found that silencing those neurons that continued to express Tac1 in adulthood resulted in a highly significant reduction in responses to a cold stimulus, and a moderate reduction in the Hargreaves test, but did not apparently alter chloroquine-evoked itch. A possible explanation for these discrepancies might involve different effects of ablation versus silencing. However, this seems unlikely, and Foster et al (2015) found very similar behavioural phenotypes following ablation or synaptic silencing of a different neuronal population (glycinergic inhibitory interneurons). An alternative explanation might be differences in the populations that were targeted in each case. Because our silencing strategy depended only on expression of a single recombinase (Cre), we may have affected SP-expressing primary afferents. However, we have found that intraspinal injection of AAV serotype 1 (as used in our initial TeLC experiments) does not result in retrograde transport from axons, and does not label SP-expressing primary afferents (Gutierrez-Mecinas et al., 2018). We were also able to confirm in the later experiments (in which we used an AAV that coded for both TeLC and eGFP) that there was no detectable labelling of primary afferent neurons in dorsal root ganglia. It is therefore unlikely that our behavioural findings resulted from silencing of peptidergic nociceptors. We have shown that there is a population of dorsal horn neurons that are captured in a reporter cross, but not by the intraspinal injection method in the Tac1^Cre^ mouse, and which therefore probably represent cells that transiently express Tac1 (Gutierrez-Mecinas et al., 2017). Ablation of these cells in the study by Huang et al may have led to the reduction in pruritogen-evoked itch. However, it seems unlikely that the inclusion of these cells in the strategy of Huang et al would result in the lack of altered nocifensive responses. Interestingly, the method used by Huang et al did not completely eliminate Tac1+ cells (88% depletion) and they did report insignificant trends towards increased latency in their Hargreaves and cold tests. It is therefore possible that we were able to silence a higher proportion of neurons, and that this difference resulted in the greater reduction in these nocifensive reflex behaviours.

Our approach will have silenced both interneurons (most of which are excitatory) and projection cells in the SDH, as well as some neurons in deeper laminae, and it is not possible to determine which of these cell types are responsible for the behavioural effects that we observed. Only around 1% of SDH neurons are projection cells (Todd, 2010) and we have found that ∼20% of neurons in this region are labelled by intraspinal injection of Cre-dependent constructs in Tac1^Cre^ mice (Gutierrez-Mecinas et al., 2017). We therefore estimate that the Tac1-expressing lamina I ALT cells are outnumbered ∼50:1 by Tac1 interneurons in this region. However, since the ALT cells are located downstream of the interneurons, silencing these may have a disproportionate effect. Subsequent studies that selectively target either the interneurons or the projection neurons will be required to address this question.
